# Supplementary material for: Optimising Vaccine Dose in Inoculation against SARS-CoV-2, a Multi-Factor Optimisation Modelling Study to Maximise Vaccine Safety and Efficacy
Source: Vaccines (Basel). 2021 Jan 22;9(2):78. doi: 10.3390/vaccines9020078 (PMC7911627; doi:10.3390/vaccines9020078)
Supplement: Supplementary file 1 [file vaccines-09-00078-s001.pdf]

Supplementary

S1. Peaking vs Saturating

We calibrated both the sigmoid function and a representative peaking curve as we have previously described in [8], and calculated AIC (Akaike Information Criterion) of both. As the difference in AIC between the models was less than 2.0, there was not considered to be evidence to choose the peaking curve over the saturating curve. Thus, the saturating (sigmoidal) curve was assumed to describe the dose-seroconversion dynamics.

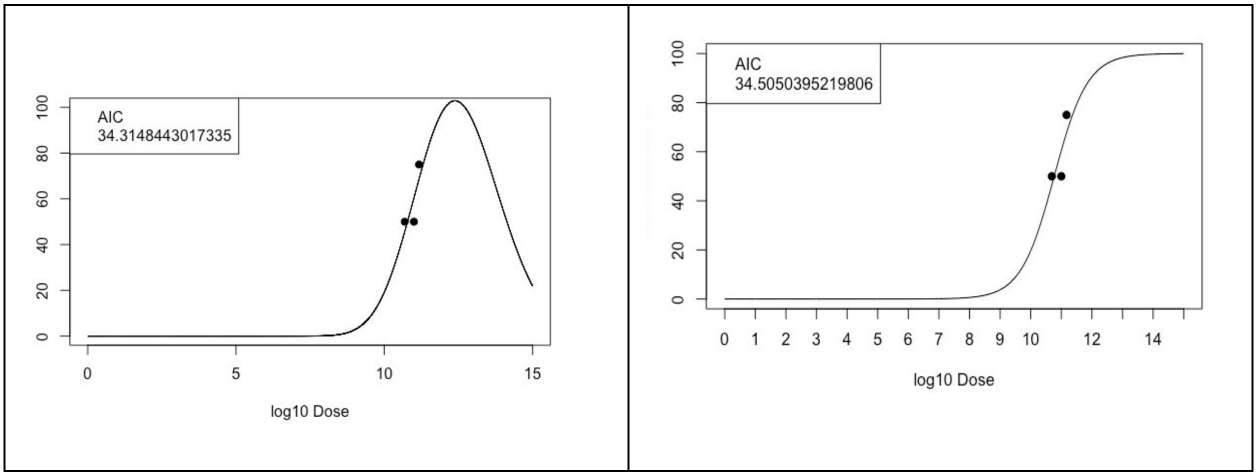

Figure S1. Calibrated peaking (left) and saturation/sigmoidal(right) curves. The y axis is predicted seroconversion, and black points are the available data. AICs are given, and were within 2 of each other.

S2. Sensitivity

We attempted to account for uncertainty in the data and models.

S2.1. Distribution of parameters

Available seroconversion data gave the number of individuals per dosing group and the number that was seroconverted. Following a bayesian perspective of the data and a parametric bootstrapping approach, we consider each dosing group as being sampled from a binomial distribution with n = 36 and the unknown true probability of seroconversion p. The likelihood distribution of p was calculated for each of the dosing groups, and we can consider that the true probability of seroconversion follows these likelihood distributions for each group. This was repeated for the adverse event data (Figure S2).

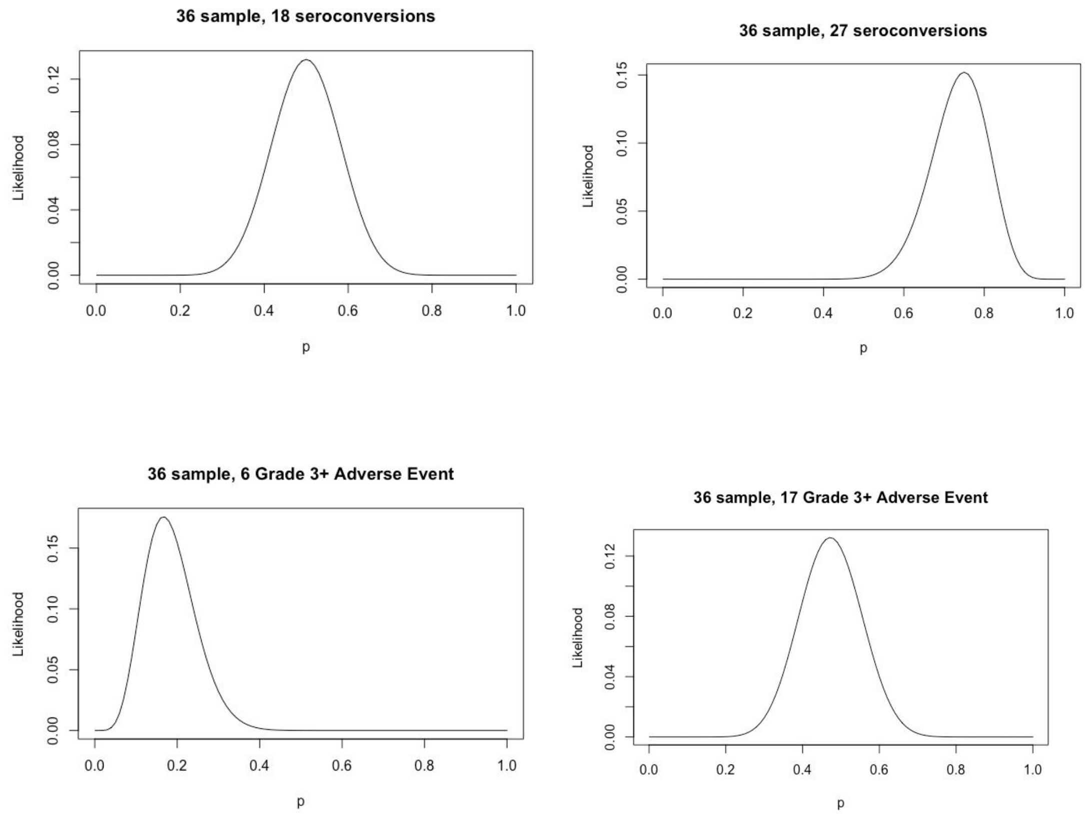

Figure S2. Likelihood distribution of true seroconversion (top) or true grade 3+ adverse reaction (bottom) probabilities for binomial processes with 36 trials and S successes. Top left shows the distribution given S = 18 (dose = 5×10^{10}, 1×10^{11}), top right shows the distribution given S = 27

(dose = 1.5 x 10^{11}), bottom left shows the distribution given S = 6 (dose = 5 x 10^{10}, 1 x 10^{11}), bottom left shows the distribution given S = 17 (dose = 1.5 x 10^{11}).

We sampled from each of these likelihood distributions 5000 times to create 5000 bootstrap dose-response data sets. For each of these data sets, we calibrated a sigmoid curve and recorded *MaxResponse, Scale,* and $Dose_{50}$ for each. This gave a distribution of the values of *MaxResponse, Scale,* and $Dose_{50}$ for the seroconversion that were reasonable giving the observed data. This was repeated for the adverse event data to give a distribution of the values of *Scale* and $Dose_{50}$ for the safety curve (Figure S3).

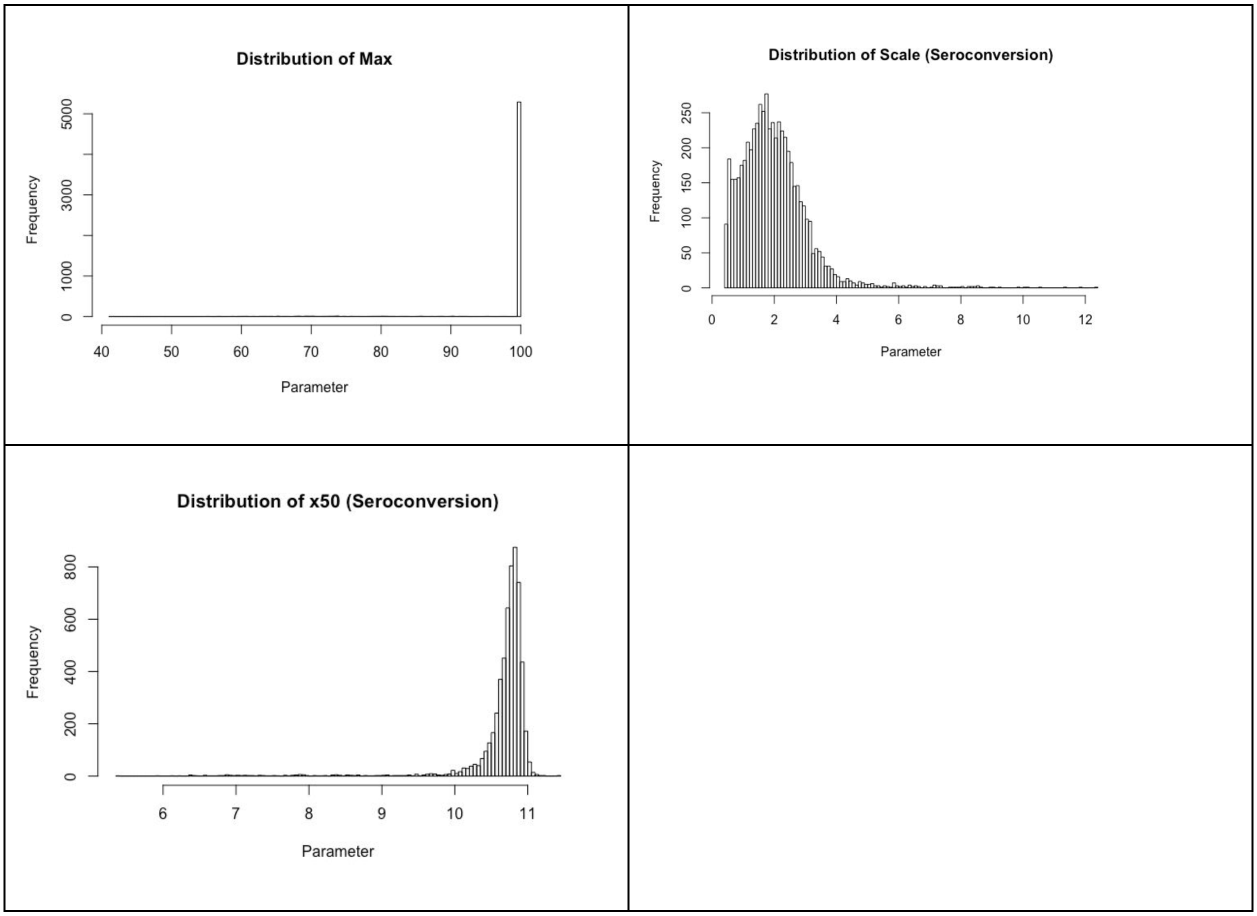

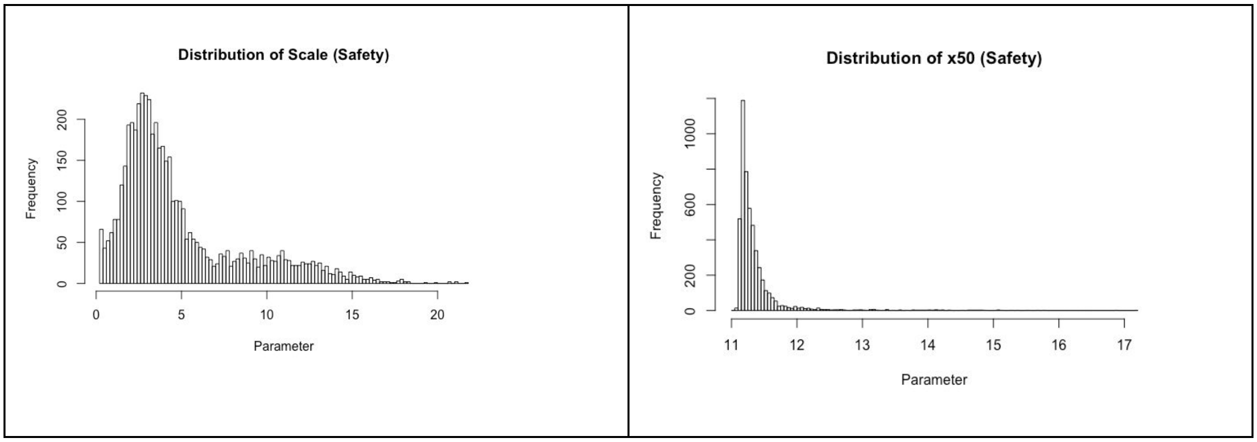

Figure S3. Distribution of model parameters following bootstrapping process with 5000 samples.

The two *Scale* parameters appeared to be the least well identified, and *MaxResponse* appeared well identified. We calculated a non-parametric 95% confidence interval for parameters by finding the 2.5th and 97.5th percentile of the parameter distributions. This is given in the varying range column of Table S1. As the parameters of the cost model were literature derived, we instead allowed them to vary in a 10% region around the value derived from literature. We write *Cost per* 10^{11} *viral particles* rather than *Cost per viral particle* due to the small size of the *Cost per viral particle* parameter. Note that this does not alter the analysis.

Parameters	Model	Description	Value	Varying range
MaxResponse	Seroconversion	Predicted seroconversion given infinite dose.	100	[70.17,100]
Scale (Seroconversion)	Seroconversion	Gradient of seroconversion sigmoid function	1.83145	[0.53, 4.31]
$Dose_{50}$ *(Seroconversion)*	Seroconversion	Dose required to reach 50% of *MaxResponse* seroconversion	10.767948	[9.49,10.97]
Scale (Safety)	Safety	Gradient of safety sigmoid function	3.76339	[0.67, 14.17]

$Dose_{50}$ (Safety)	Safety	Dose required to reach 50% of individuals experiencing grade 3+ adverse events	11.6145	[11.12, 12.35]
$Cost_{Delivery}$	Cost	Dose independent vaccination costs	5.24	[4.72, 5.77]
Cost per 10^{11} viral particles	Cost	Dose dependent vaccination costs (per 10^{11} VP)	0.76	[0.68, 0.84]

Table S1. Parameters that were explored through sensitivity analysis. The 'value' column gives the numerical value which were used in objectives 2 and 3 and the 'varying range' column gives a reasonable bound on the parameter values as in supplementary S2.1.

S2.2. Parameter Sensitivity

These parameters define the utility function. To determine the sensitivity of the optimal dose prediction to a parameter, θ, we fix all other parameters at the calibrated/literature derived value and allow θ to vary in the region around it that we just defined [Table S1].
The optimal dose for each of these varying θ values were calculated and plotted. We did this analysis for both the costless and cost utility functions.

S2.2.1. Costless

The utility function was most sensitive to variance in the *$Dose_{50}$ (Seroconversion)* and *Scale (Safety)* parameters, but some uncertainty in optimal dose may also be caused by variance in the estimated *Scale (Seroconversion)* parameter.

MaxResponse

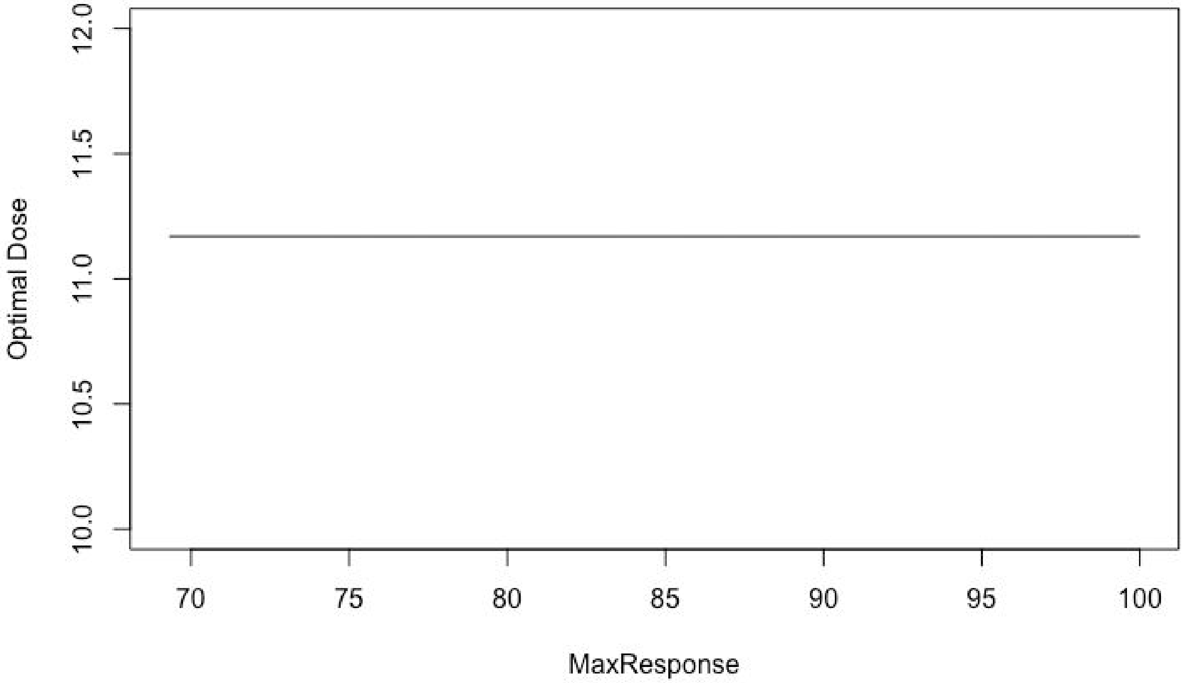

Scale (Seroconversion)

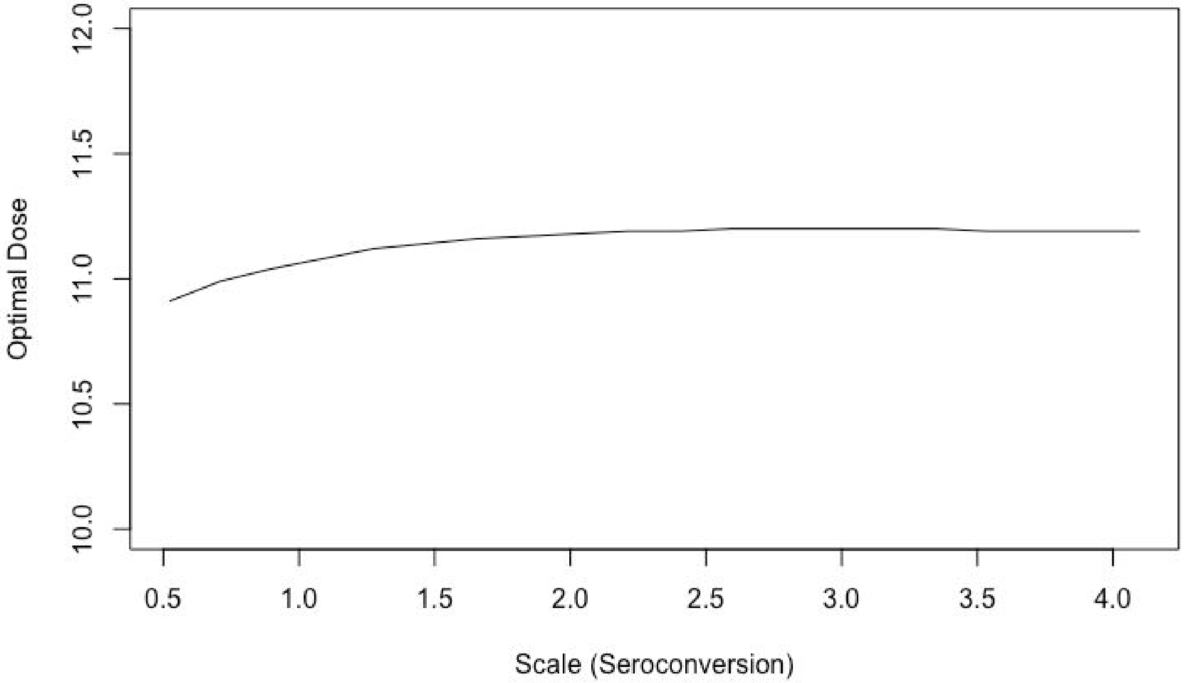

$Dose_{50}$ (Seroconversion)

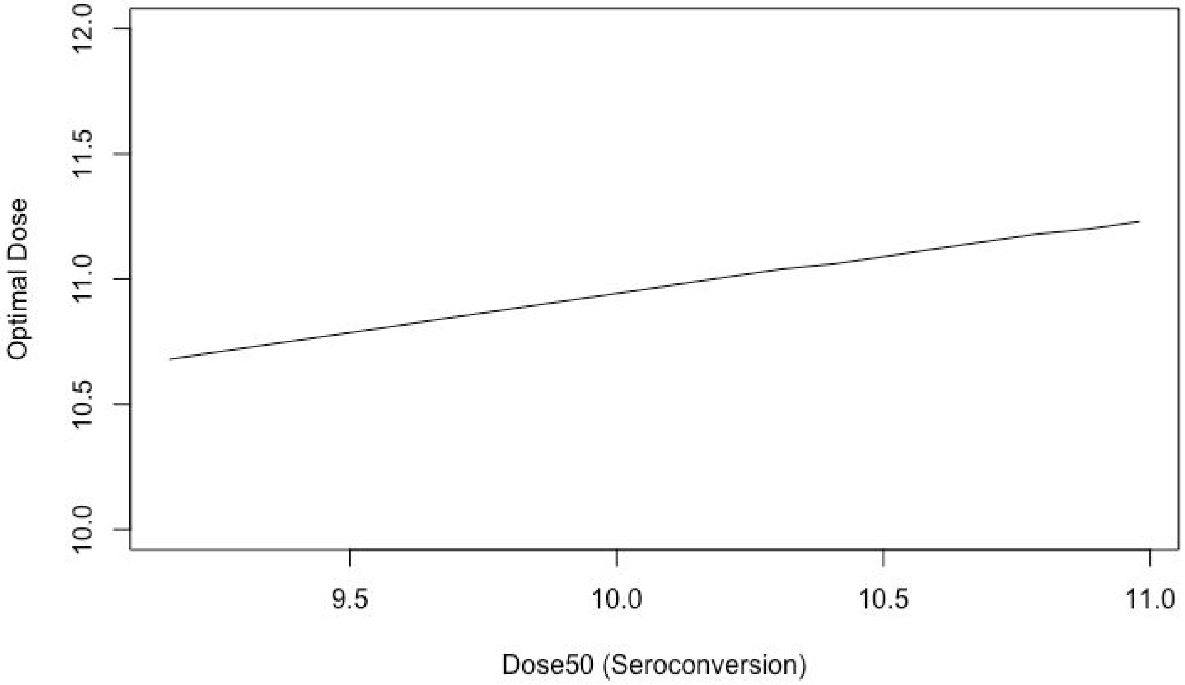

Scale (Safety)

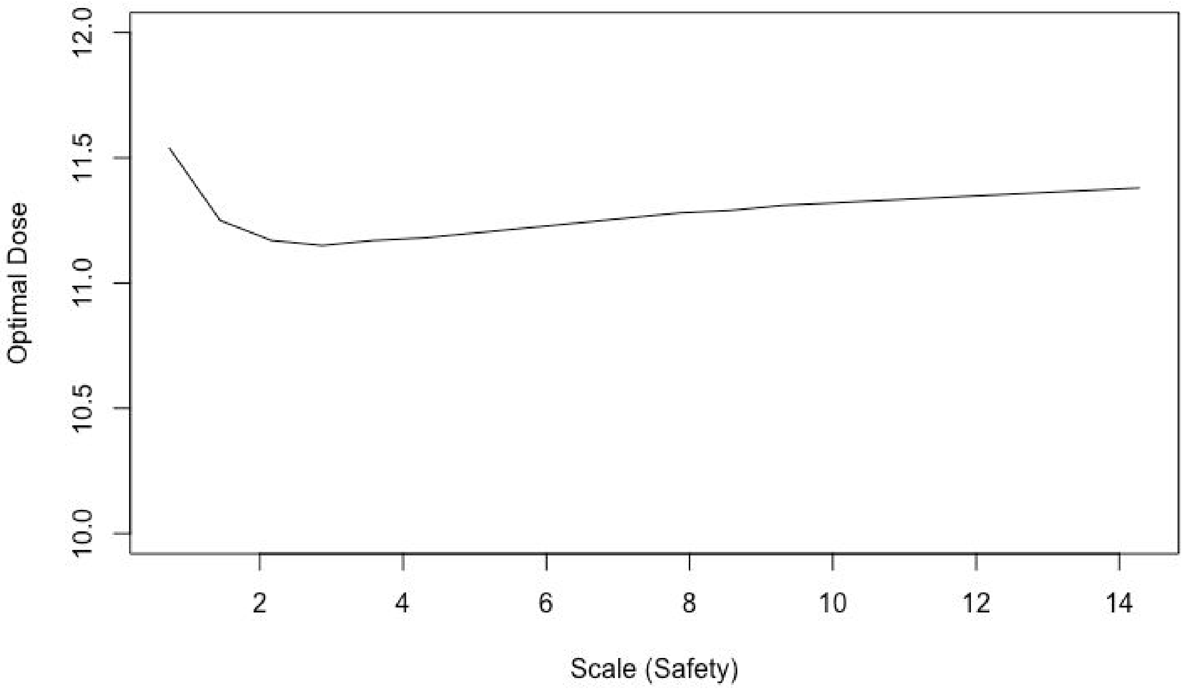

$Dose_{50}$ (Safety)

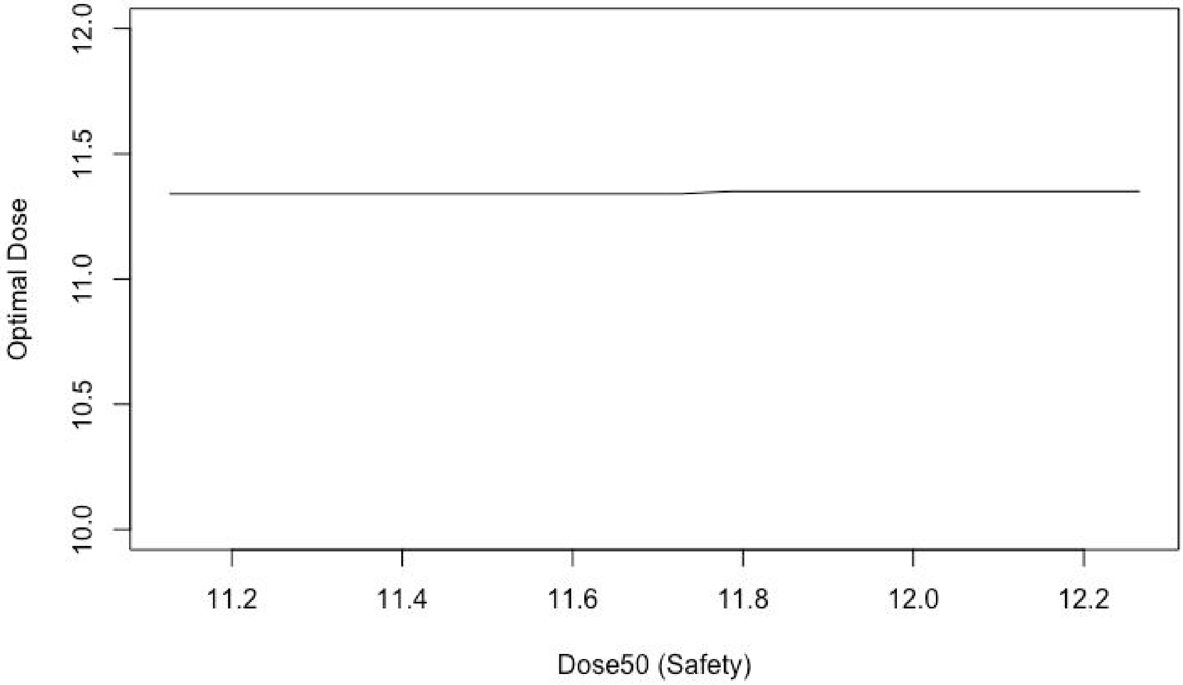

Optimal Dose
12.0
11.5
11.0
10.5
10.0
11.2
11.4
11.6
11.8
12.0
12.2
Dose50 (Safety)

S2.2.2. Costed

The utility function was again most sensitive to variance in the *Dose*$_{50}$ *(Seroconversion)* parameter, but was less sensitive to uncertainty in the *Scale (Safety)* parameter. Again, optimal dose may also be caused by variance in the estimated *Scale (Seroconversion)* parameter. Optimal dose did not appear to be sensitive to variance in either cost parameter.

MaxResponse

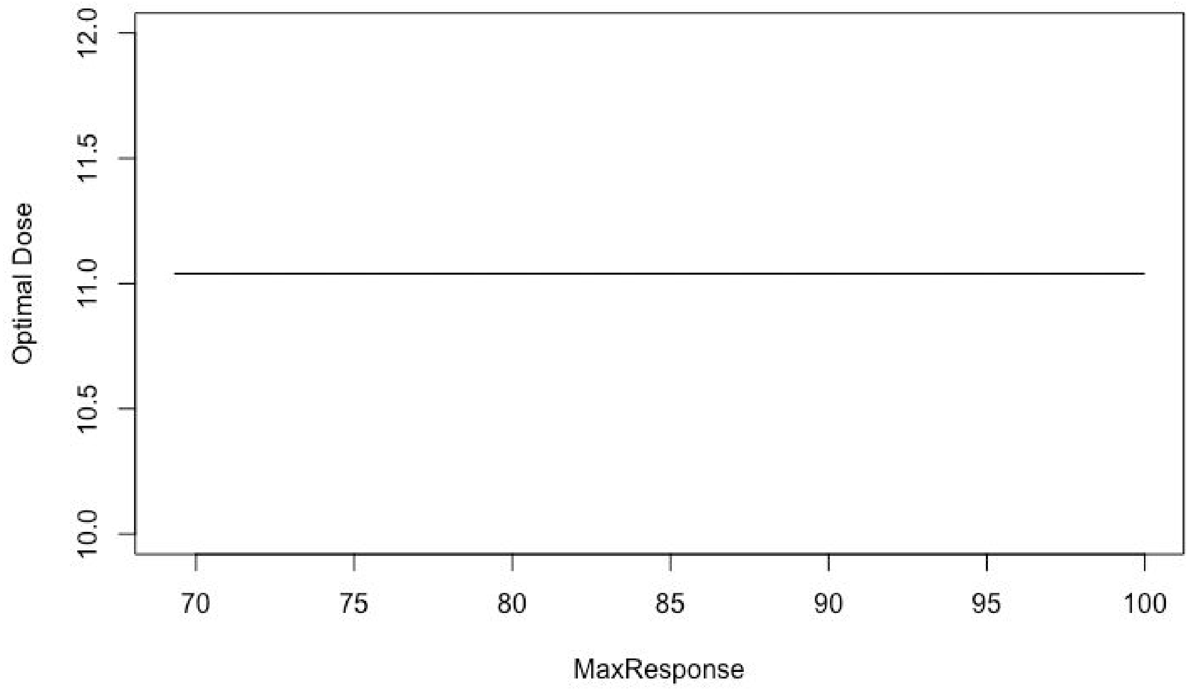

Scale (Seroconversion)

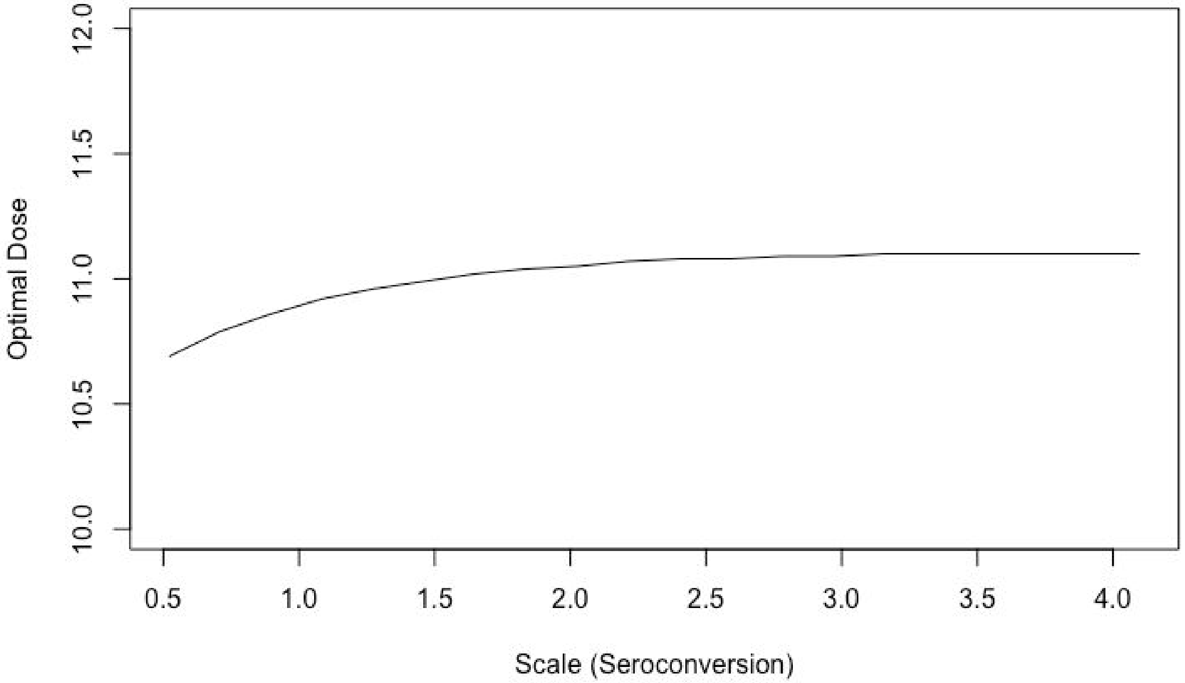

$Dose_{50}$ (Seroconversion)

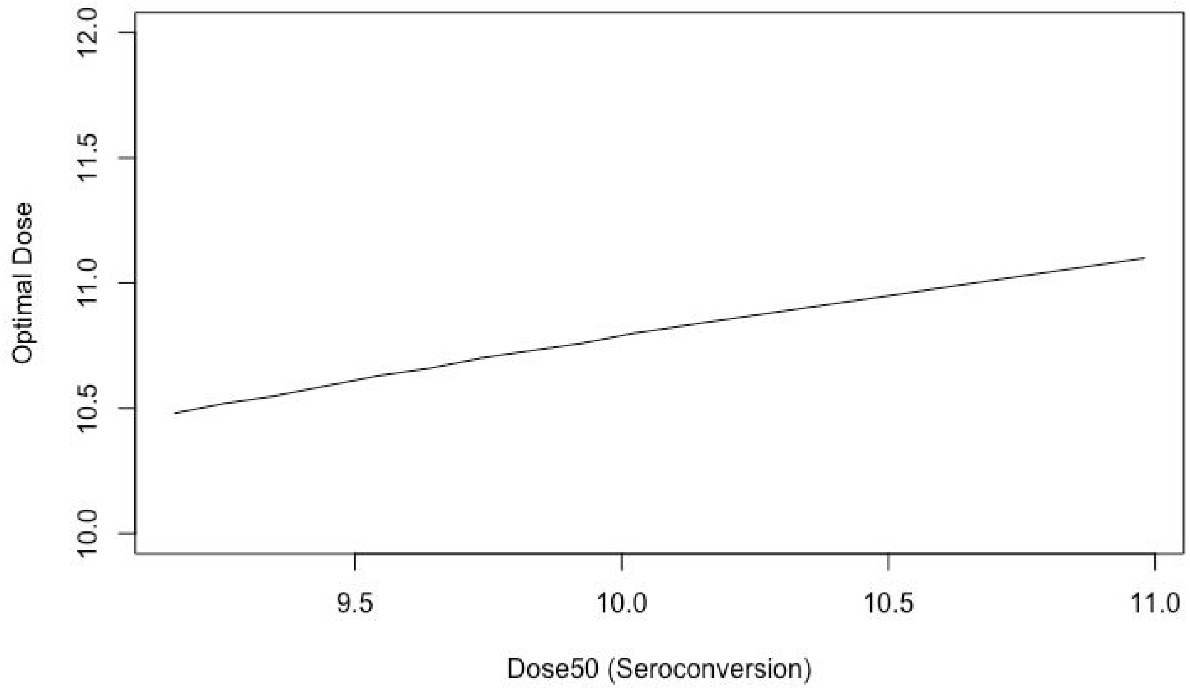

Scale (Safety)

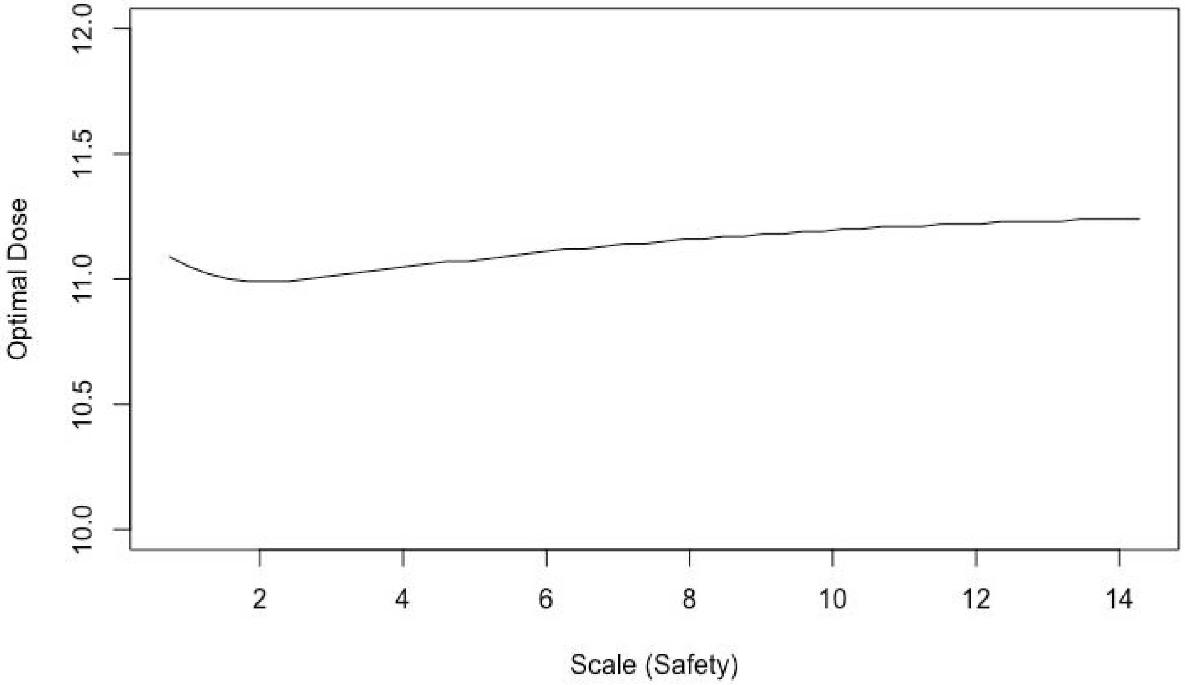

$Dose_{50}$ (Safety)

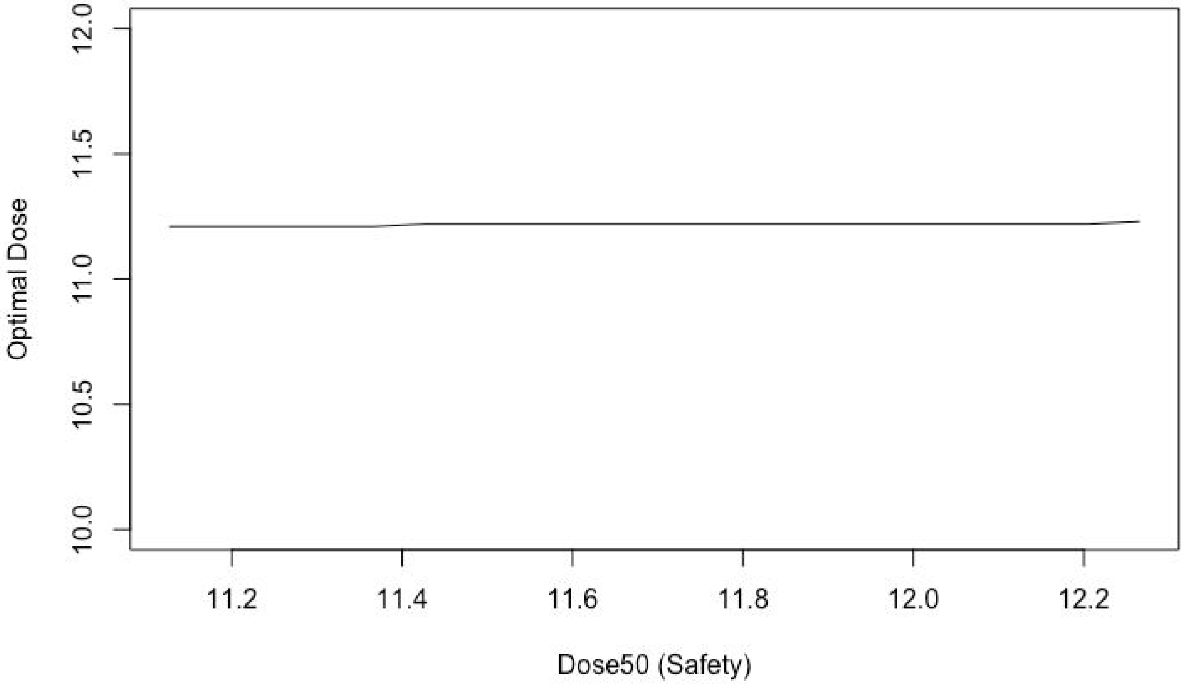

$Cost_{Delivery}$

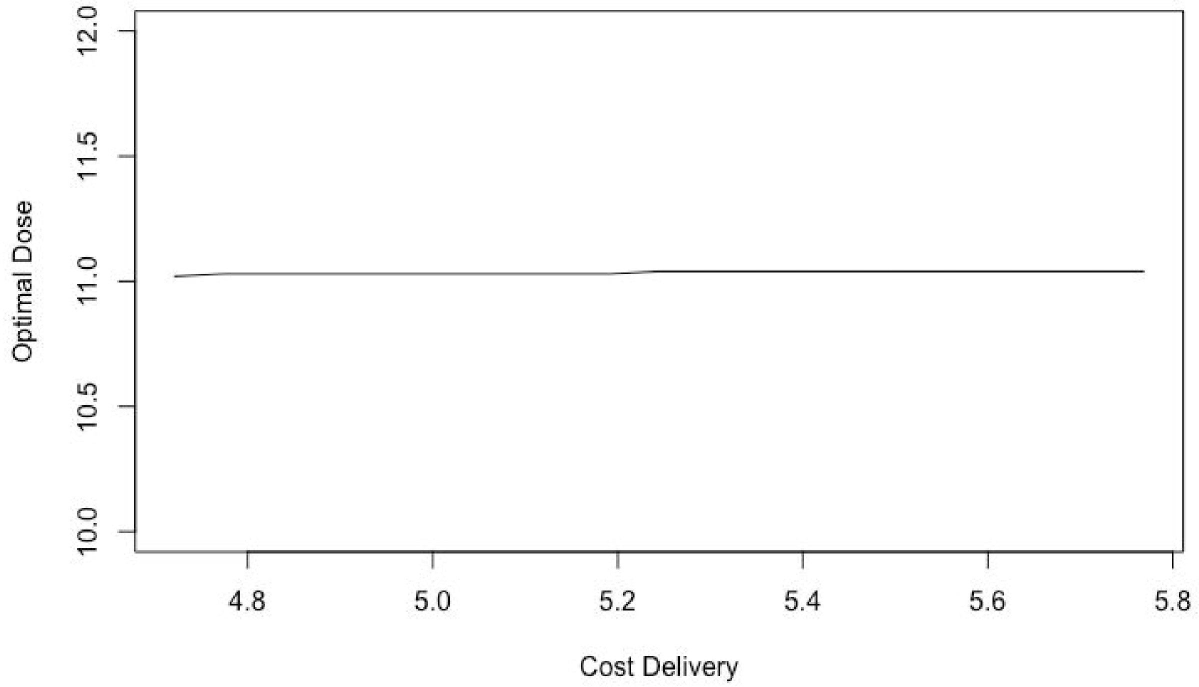

Cost per 10^{11} viral particles

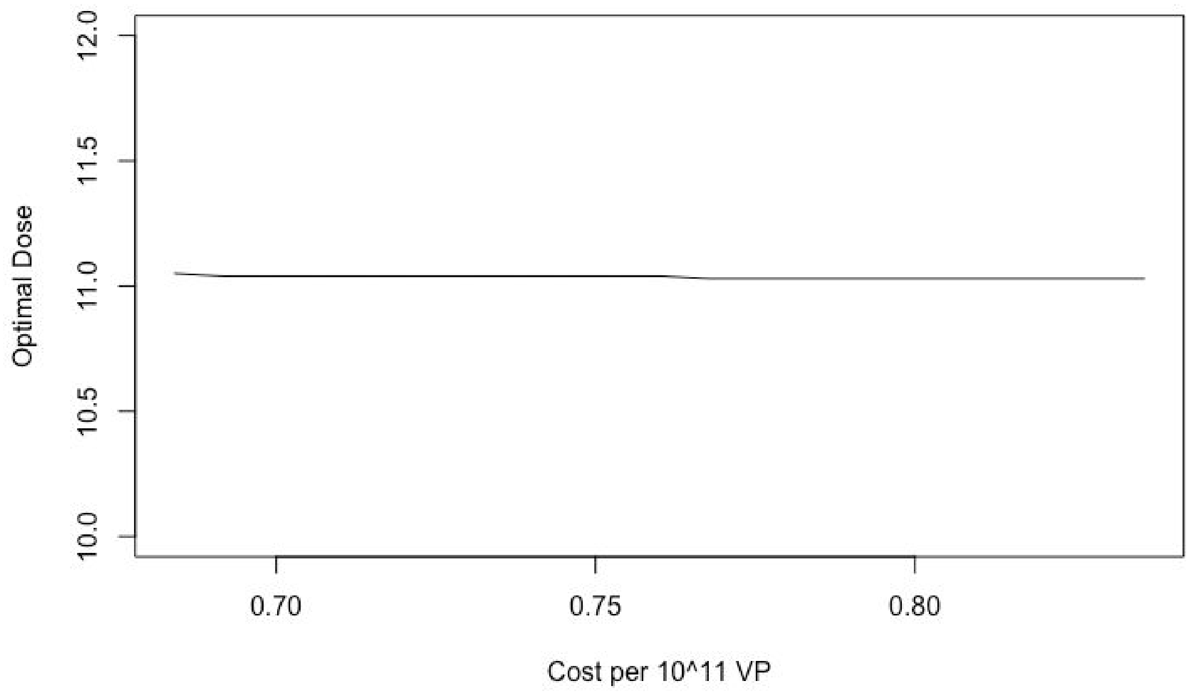

S2.3. Optimal dose Confidence Interval

We resampled with replacement from the bootstrap dose-response data calibrated parameter sets. We did this for a combined 10,000 seroconversion/safety parameter sets. For these we calculated the optimal dose as defined by all utility functions. This was used to calculate an approximate 95% confidence interval for the optimal dose of both utility functions.

S2.3.1. Herd Immunity

Optimal dosing follows an approximate skewed normal distribution (figure S4), with the qualitative peak being approximately $10^{11.11}$ (=1.3 x 10^{11}). The calculated 2-tailed 95% distribution of the data had lower bound $10^{10.90}$ (= 0.8 x 10^{11}) and upper bound $10^{11.90}$ (= 7.9 x 10^{11}).

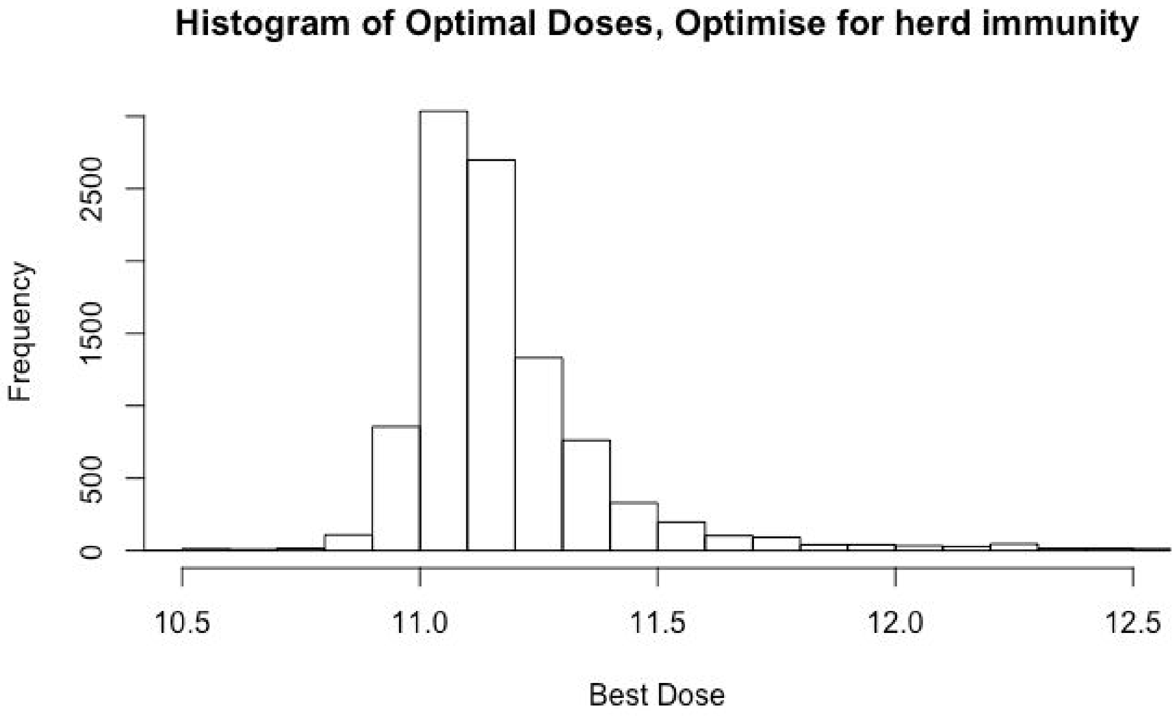

Figure S5. Distribution of optimal dose based on herd immunity from parametric bootstrapping of the data.

S2.3.2. Costless

Optimal dosing follows an approximate normal distribution (figure S4), with the qualitative peak being approximately 10^{11} (=1.0 x 10^{11}). The calculated 2-tailed 95% distribution of the data had lower bound $10^{10.46}$ (= 0.29 x 10^{11}) and upper bound $10^{11.67}$ (= 5.0 x 10^{11}).

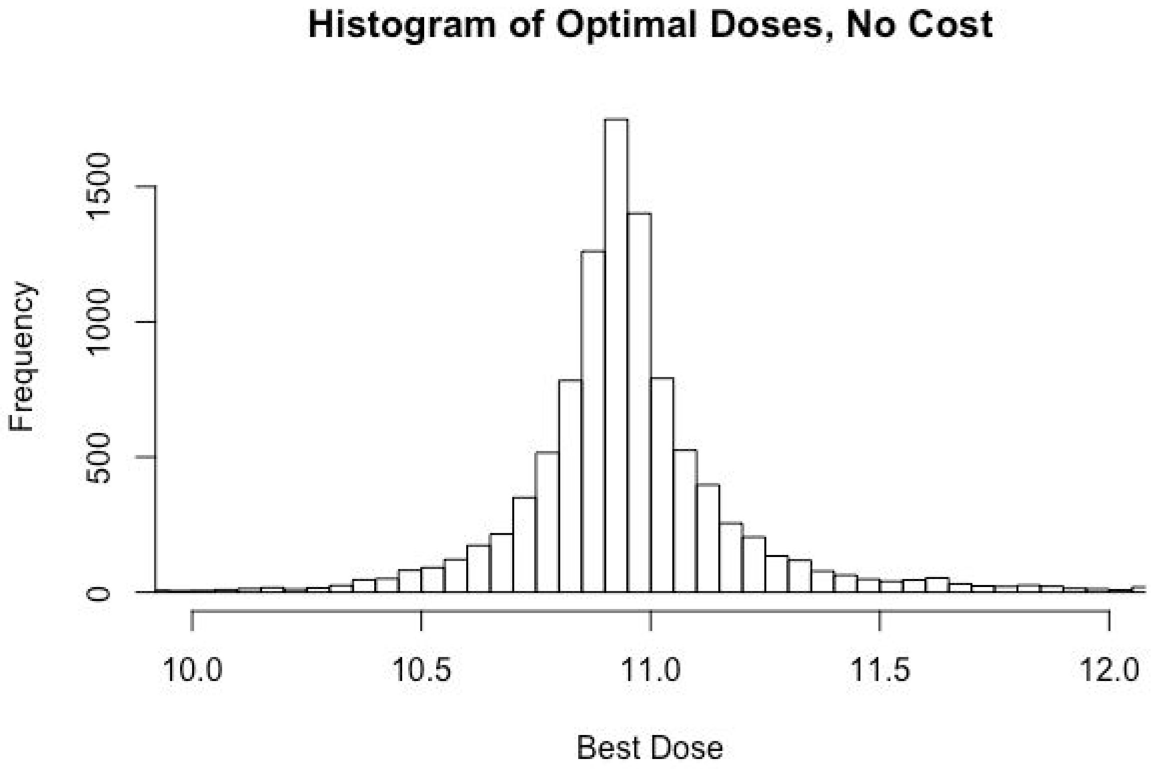

Figure S6. Distribution of optimal dose without including cost from parametric bootstrapping of the data.

S2.3.3. Costed

Optimal dosing follows an approximate left-skewed normal distribution (figure S5), with the qualitative peak at approximately $10^{10.9}$ (= .75 x 10^{11}). The calculated 2-tailed 95% distribution of the data had lower bound $10^{10.32}$ (= 0.21 x 10^{11}) and upper bound $10^{11.18}$ (= 1.54 x 10^{11}).

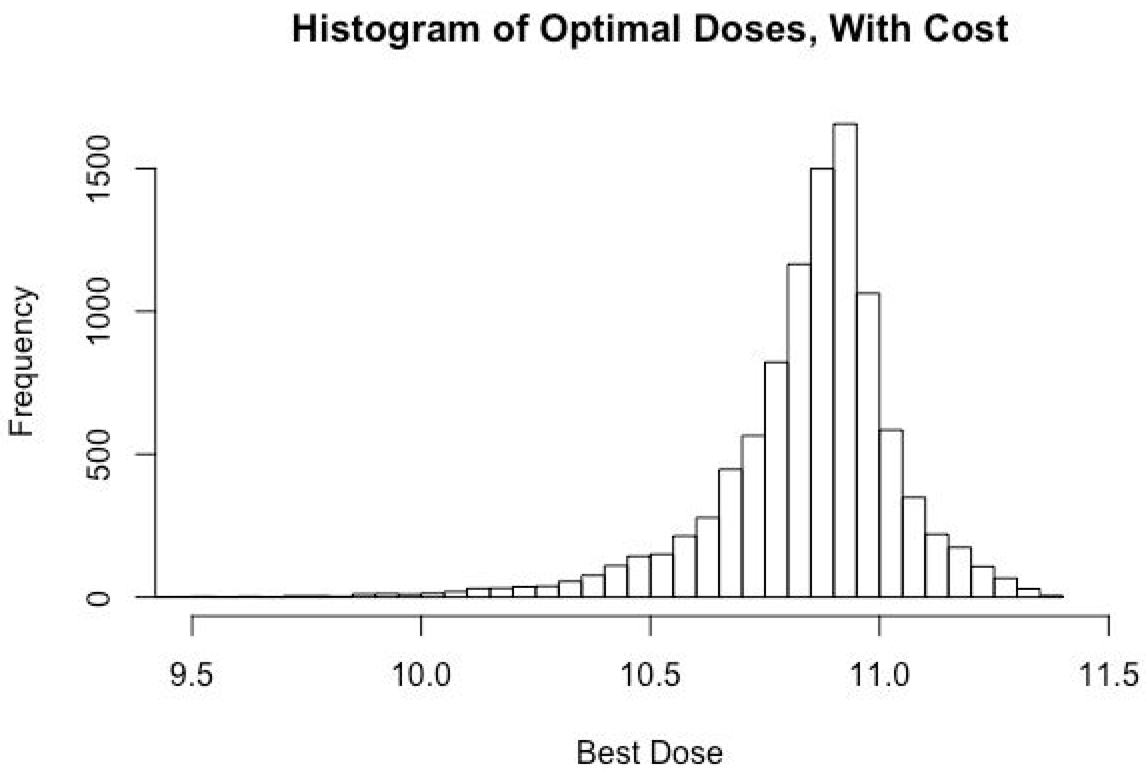

Figure S7. Distribution of optimal dose (log10 scale) including cost from parametric bootstrapping of the data.

S3. Population demographics

Population demographics of age, gender, and pre-existing adenovirus neutralising antibody titre as described in the body of work the data were extracted from [15].

	Dose (VP)		
	5.0 x10^{10}	1.0 x 10^{11}	1.5 x 10^{11}
Age			
18–29	9 (25%)	12 (33%)	10 (28%)
30–39	13 (36%)	14 (39%)	15 (42%)
40–49	8 (22%)	3 (8%)	7 (19%)
50–60	6 (17%)	7 (19%)	4 (11%)
Mean age (years)	37.2 (sd =10.7)	36.3 (sd = 11.5)	35.5 (sd = 10.1)
Sex			
Male	18 (50%)	19 (53%)	18 (50%)
Female	18 (50%)	17 (47%)	18 (50%)
Pre-existing adenovirus type-5 neutralising antibody			
≤200, titre	16 (44%)	17 (47%)	20 (56%)
>200, titre	20 (56%)	19 (53%)	16 (44%)
Mean geometric mean titre	168.9 (13.9)	149.5 (10.5)	115.0 (13.4)

Table S2. Distribution of sample covariates for each dosing group. Data are given as number (percentage).

S4. Variability in the data.

We note that in plot 2b the data shows that for the three dosing groups (5.0×10^{10}, 1.0×10^{11} and 1.5×10^{11}), 86%, 83%, and 75% of individuals experienced any grade adverse events respectively. This represents respectively that for each of the three dosing groups of size N=36 (31,30, and 27) individuals of individuals experienced any grade adverse events. There is a qualitative downwards trend, which our strictly-increasing sigmoid model would be unable to model.

We considered this data using whilst taking the interpretation that individuals are independent samples of an underlying Bernoulli process we can calculate the 95% confidence interval on the true probability of experiencing any grade adverse events, using a similar approach to that described in [S2]. These are:

a) For dose 5.0×10^{10}; 86% (71%,95%)
b) For dose 1.0×10^{11}; 83% (67%,94%)
c) For dose 1.5×10^{11}; 75% (58%,88%)

As these confidence intervals do overlap, we did not believe that there was sufficient justification to consider the possibility that an increased dose could reduce the number of adverse events experienced, even given the downward trend observed. We believe it more likely that all three data points have approximately similar probabilities of any grade adverse events.

To illustrate this point please consider the below plot, which shows the data described overlaid with the 95% confidence intervals for Bernoulli trials assuming that our underlying model is correct. As all of the points are within these bounds, again this model seems reasonable with the

available data.

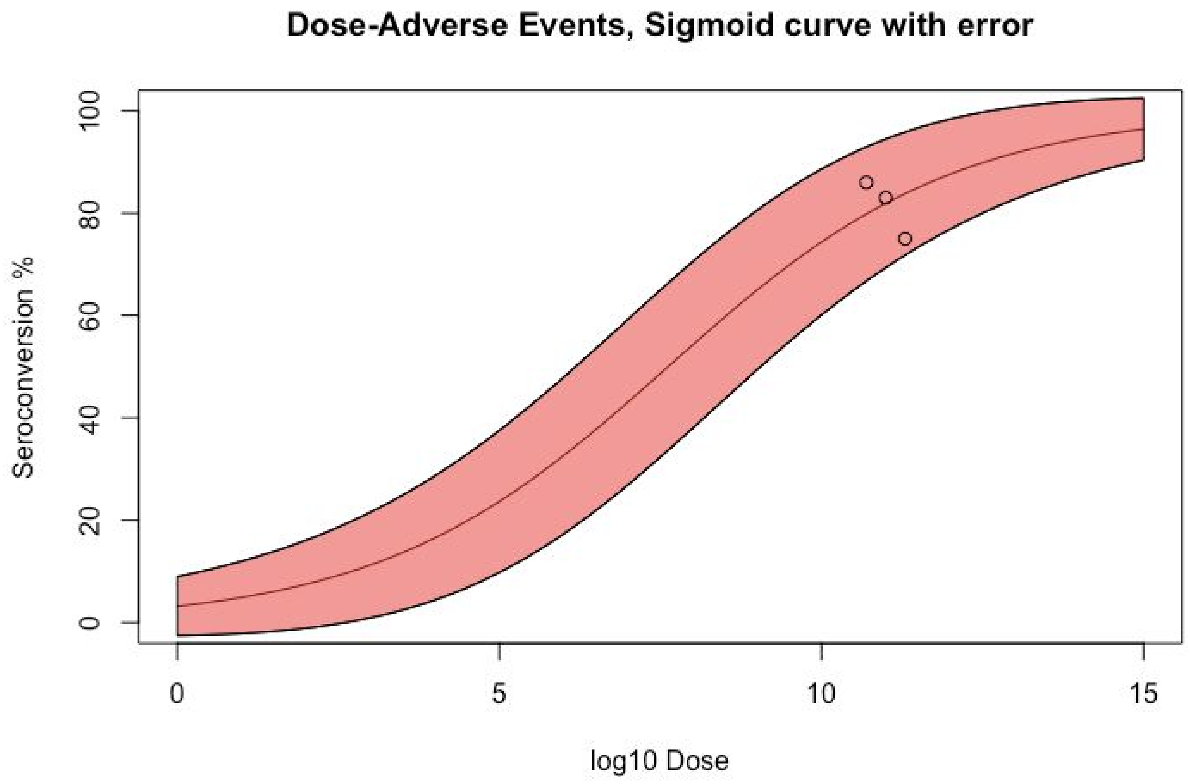

Figure S8. A plot of the expected any-grade adverse event data compared to observed data The black line plots the calibrated curve. The red area plots the 95% confidence interval for the percentage of individuals that would experience any-grade adverse events in a 36-per-group trial assuming that this is the true model (for example, if the true probability of an adverse event for a given dose is 0.5, then approximately 95% of trials of that dose with size 36 would have between 13(=36%) and 23(=63%) individuals experiencing adverse events)

However, further investigation into the relationship between dose and proportion of individuals experiencing adverse events would be useful if there was sufficient data.

S5. Threshold analysis, Bivariable

We considered varying both of the $Cost_{Delivery}$ and *Cost per* 10^{11} *viral particles* parameters in the +/- 3 orders of magnitude range at the same time, and found that for high values of $Cost_{Delivery}$ the optimal dose was independent of *Cost per* 10^{11} *viral particles* (Figure S6). If these plots are censored to include only points where the predicted optimal dose is less than 10^{11}, 5 x 10^{11} and 10^{10} VP, we find the behaviour observed in Figures S7, S8 and S9 respectively. A clear linear separation is observed for all three plots. By finding the line between (0,0) and the point with the maximum $Cost_{Delivery}$, we can approximate these decision boundaries as respectively

10^{11}: *Cost per* 10^{11} *viral particles*= 0.2 x $Cost_{Delivery}$

5 x 10^{10}: *Cost per* 10^{11} *viral particles*= 1.3 x $Cost_{Delivery}$

10^{10}: *Cost per* 10^{11} *viral particles*= 17.9 x $Cost_{Delivery}$

Hence, we can suggest that, assuming no uncertainty in the safety and seroconversion related model parameters;

If the cost per 10^{11} VP is greater than 0.2 times the cost per vaccination that is independent of dose, optimal dose is less than 10^{11} VP.

If the cost per 10^{11} VP is greater than 1.3 times the cost per vaccination that is independent of dose, the optimal dose is less than 5 x 10^{10} VP.

If the cost per 10^{11} VP is greater than 17.9 times the cost per vaccination that is independent of dose, the optimal dose is less than 10^{10} VP.

In all other cases optimal dose is greater than 10^{11}, with the largest recommended dose across all costing parameters was 1.5 x 10^{11} VP, which is the dose recommended by the results in objective 2.

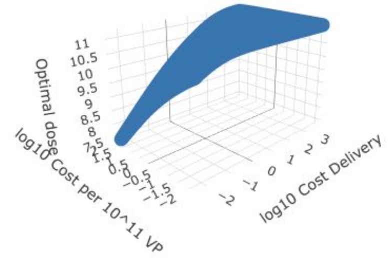

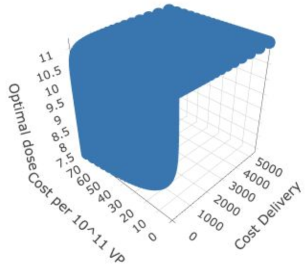

Figure S9. Optimal predicted dose for +/- 3 orders of magnitude (log10 scale) around *Cost per 10^{11} viral particles* and $Cost_{Delivery}$.The left has *Cost per 10^{11} viral particles* at a log10 scale and the right scaled normally.

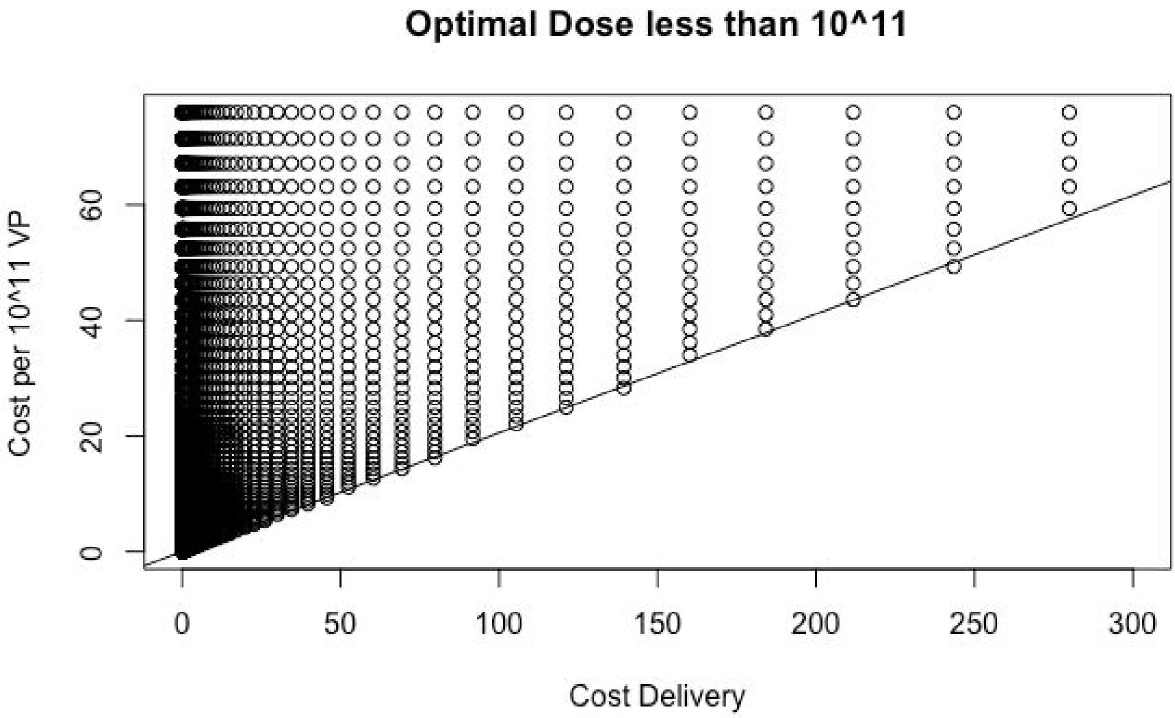

Figure S10. Pairs of *Cost per 10^{11} viral particles* and $Cost_{Delivery}$ for which the optimal predicted dose was less than 10^{11} VP. Black line represents the estimated decision boundary.

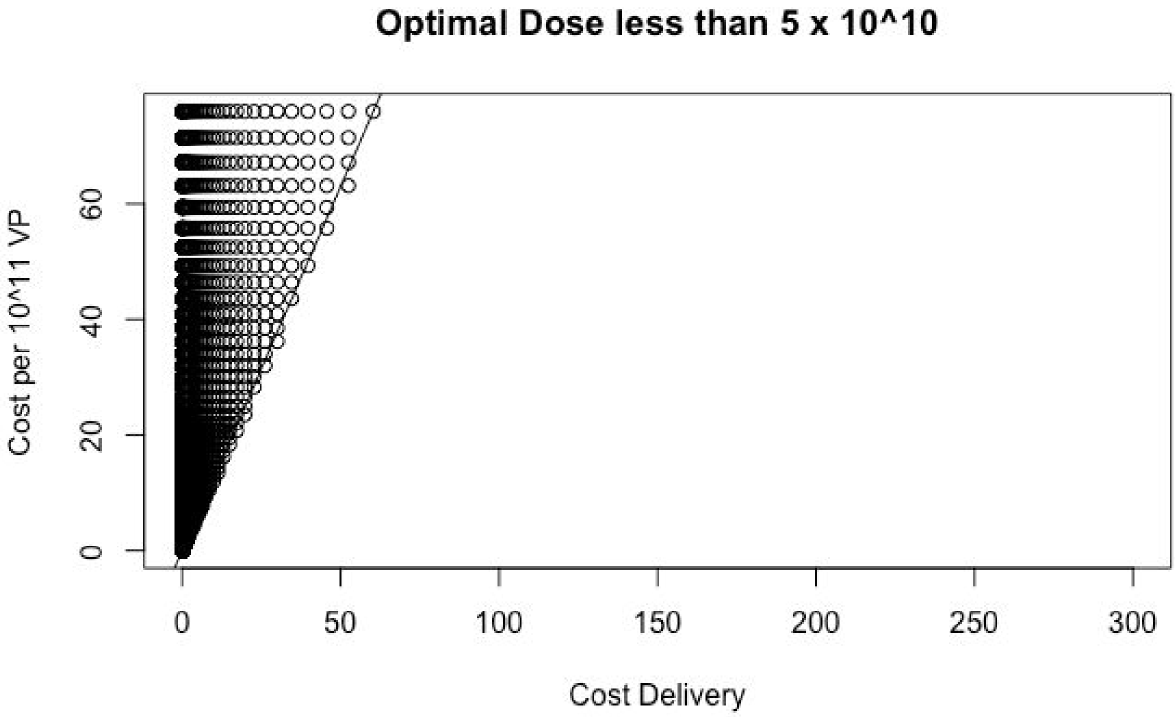

Figure S11. Pairs of *Cost per* 10^{11} *viral particles* and $Cost_{Delivery}$ for which the optimal predicted dose was less than 5 x 10^{10} VP. Black line represents the estimated decision boundary.

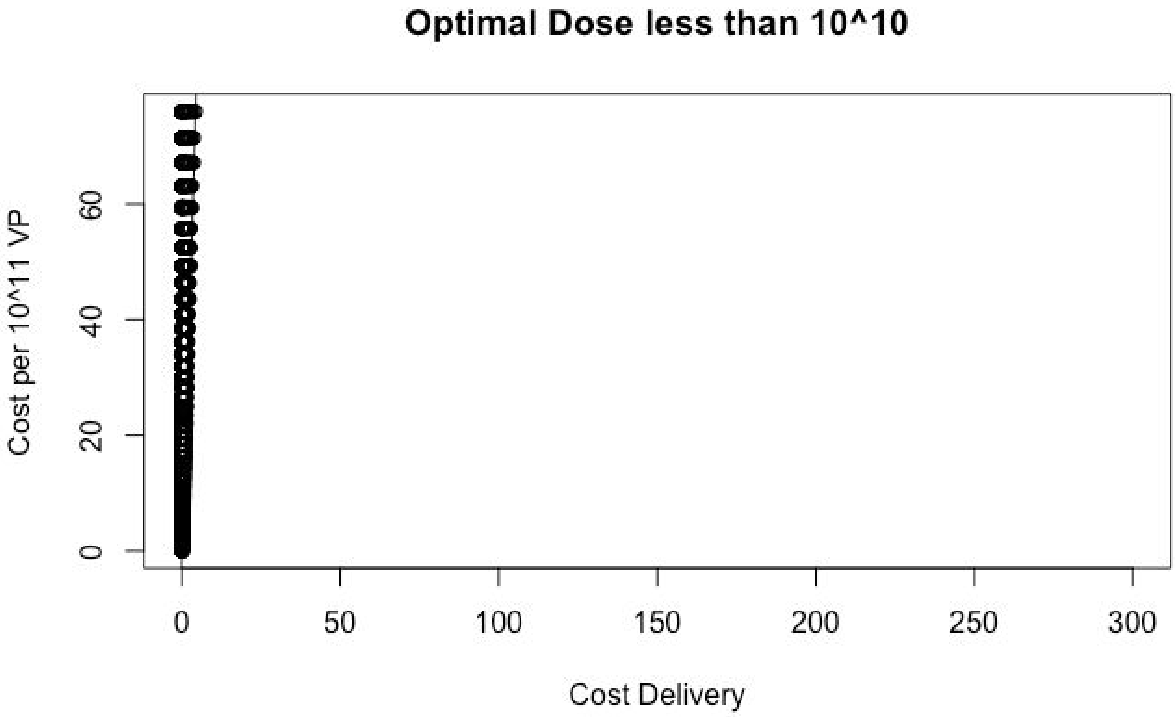

Figure S12. Pairs of *Cost per* 10^{11} *viral particles* and $Cost_{Delivery}$ for which the optimal predicted dose was less than 10^{10} VP. Black line represents the estimated decision boundary.

S6. Weighted Utility Functions

We suggested that there alternate approach to the utility function, where we weight the expected discomfort of a SARS-CoV-2 infection relative to the expected discomfort of receiving a vaccination. This approach requires defining such a weighting, which would require making additional assumptions and introducing complexity that we did not believe added to the main body of this work. Whilst establishing reasonable weightings are beyond the scope of this work, we suggest potential utility functions with pseudo-arbitrary values for the weighting. Hence, whilst these utility functions would not be useful presently for decision-making, if weights could accurately be determined they may be informative. Hence potential weighted utility functions are proposed. We note that the likely method of determining weighting is through a questionnaire of experts and decision makers or through group discussion, as is typical for determining weightings in multi-criteria decision analysis [9].

S6.1. 2:1 Ratio

The utility functions recommended in 3.4 and 3.5 assume that the only desirable outcome of vaccination is seroconversion without experiencing grade 3+ adverse events. This, implicitly, assumes that both seroconverting and avoiding grade 3+ adverse events are equally as desirable. Alternatively, we could consider all outcomes of vaccination with relative weightings of utility. We (pseudo-arbitrarily) choose a 2:1 weighting, where seroconversion is twice as desirable as avoiding a grade 3+ adverse event. The possible outcomes are namely;

- Not seroconverting or experiencing grade 3+ adverse events.
- Not seroconverting, experiences grade 3+ adverse events.
- Seroconversion, does not experience grade 3+ adverse events.
- Seroconversion, experiences grade 3+ adverse events.

The below table indicates the relative 'scores' of each of these outcomes.

Name	Experiences Seroconversion (+2)	Experience Grade 3+ Adverse Events (-1)	Score
$S'A'$	NO	NO	0 (= 0 + 0)
$S'A$	NO	YES	-1 (= 0 - 1)
SA'	YES	NO	2 (= 2 + 0)

SA	YES	YES	1 (= 2 - 1)

So defining P_S as the probability of seroconversion, $P_{S'} = 1 - P_S$ as the probability of no seroconversion, P_A as the probability of experiencing grade 3+ adverse events, $P_{A'} = 1 - P_A$ as the probability of not experiencing grade 3+ adverse events, we have the following utility function.

$$U_{2:1}(Dose) = Score(SA) \times P_s \times P_A + Score(SA') \times P_s \times P_{A'} + Score(S'A) \times P_{s'} \times P_A + Score(S'A') \times P_{s'} \times P_{A'}$$

Below is the dose-utility function for this utility function. We see that under this function and weighting the utility increases with dose, before decreasing. For sufficiently large doses the utility tends to $1 = Score(SA)$, as the model predicts 100% of individuals experience seroconversion and grade 3+ adverse events.

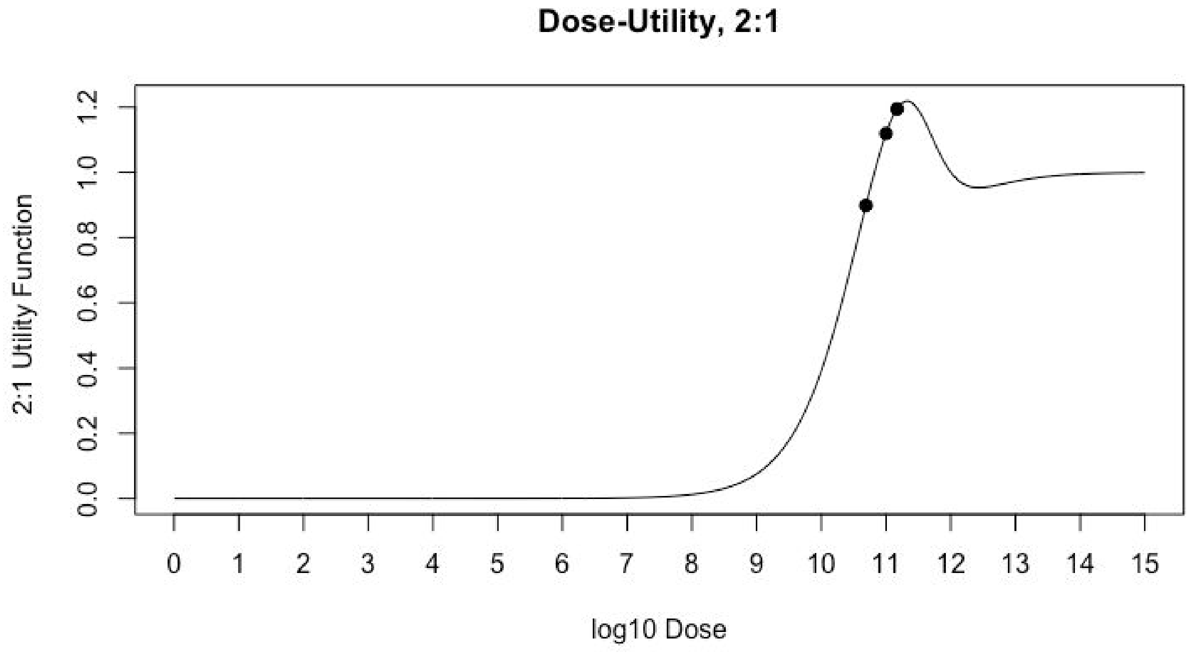

Figure S13. Dose-Utility for the 2:1 utility function. Black dots represent the empirically tested doses.

S6.2. Expected discomfort

Alternatively, we can look to expressly minimise expected discomfort. We can consider that an individual has two sources of potential discomfort, namely discomfort arising from the vaccination and discomfort arising from the disease that the vaccine aims to prevent or minimise symptoms of.

We consider that for these two sources of potential discomfort, the discomfort could be rated as Mild (Grade 1,2), Severe (Grade 3), Critical without being fatal (Grade 4 if non-fatal), Critical and Fatal (Grade 4 if fatal). The probability for each of these outcomes if an individual's contracts SARS-CoV-2 are derived from literature [43], and the probability of mild or severe adverse events are estimated from the 'dose-any grade adverse event' and 'dose-grade 3+ adverse event' models discussed in the main body of this work. As we have no data to estimate the relationship between dose and the other two outcomes, we assume that the vaccine cannot cause either of these outcomes.

These outcomes are each assigned weights for discomfort, which are not based on literature but represent the idea that critical sickness or death are significantly worse outcomes than mild sickness.

Adverse Event	Vaccine probability	SARS-CoV-2 probability	Discomfort weight (pseudo-arbitrary)
Mild	Estimated in the paper as a function of dose, any grade adverse events, P_{A1}	81%	$1 = DW_M$
Severe	Estimated in the paper as a function of dose, grade 3+ adverse events, P_{A3+}	14%	$5 = DW_S$
Critical (Non-fatal)	Assumed 0%	2.7%	$50 = DW_C$
Critical (Fatal)	Assumed 0%	2.3%	$100 = DW_F$

We can define the expected discomfort of contracting SARS-CoV-2 as

$$ExpectedDiscomfort_{Sars} = DW_M \times .81 + DW_S \times .14 + DW_C \times .027 + DW_F \times .023$$

$$ExpectedDiscomfort_{Sars} = 5.16$$

We can also estimate that an individual has a 65.5% (=0.655) (the herd immunity threshold) probability of contracting SARS-CoV-2 if they are not protected. However, this may be reduced depending on the percentage of individuals in the population that have previously contracted or received a vaccine for SARS-CoV-2 (which could be investigated by considering epidemiological models).

Hence a vaccinated individual is predicted to experience expected discomfort as a function of dose:

$$\begin{aligned} Expected\ Discomfort\ (Dose) \ = \ & P_{A1}(Dose) \times DW_M \\ & + \ P_{A3+}(Dose) \times DW_S \\ & + \ 0.655 \times P_{S'}(Dose) \times ExpectedDiscomfort_{Sars} \end{aligned}$$

Where $P_{S'}(Dose)$ is the probability of not seroconverting and hence not being protected as a function of dose. The plot below shows this relationship.

For these weights, the following behaviour is observed. As the dose increases from 0, the increasing discomfort of vaccination, whilst small, is not justified by the possible reduction in SARS-CoV-2 risk, as there is no meaningful level of seroconversion. For doses at approximately 10^{11}, we see a reduction in expected discomfort. At higher doses, whilst seroconversion is probable, the probability of grade 3+ adverse events is large enough that vaccination at this dose may be considered to be less comfortable than the average SARS-CoV-2 infection.

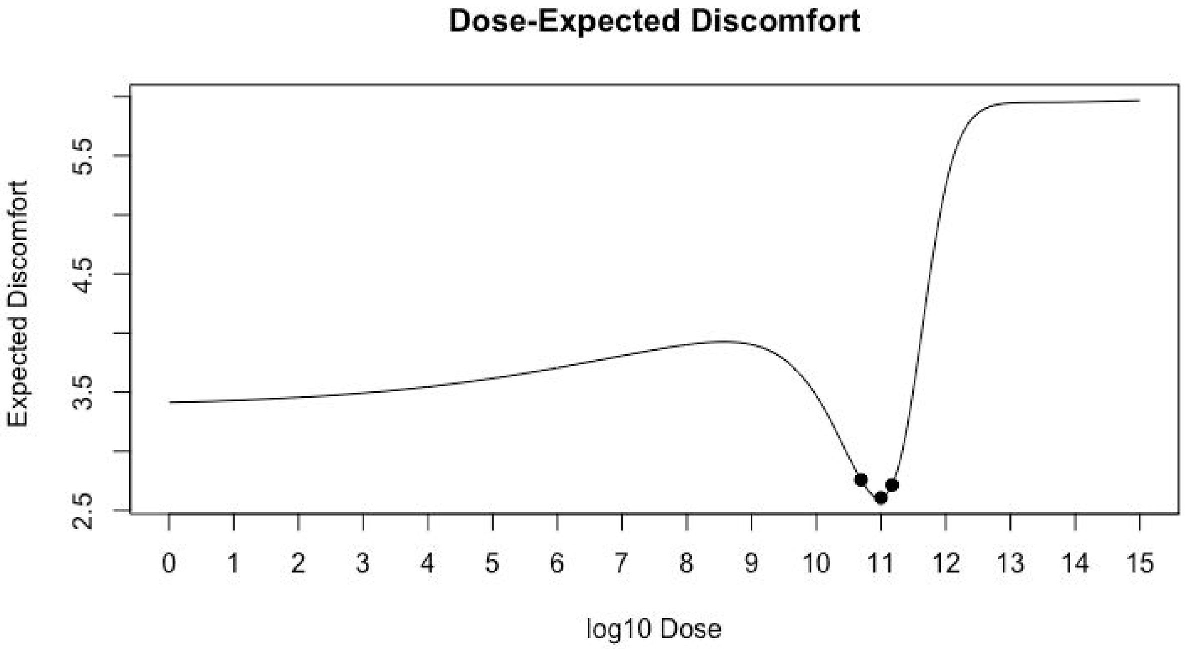

Figure S14. Dose-Utility for the expected discomfort utility function. Black dots represent the empirically tested doses.

We can also consider the expected reduction in discomfort from baseline by subtracting the dose-dependent expected discomfort from the zero-dose expected discomfort.

$$\begin{aligned} Expected\ Discomfort\ Reduction\ (Dose) = \ & 0.655 \times P_{S'}(0) \times ExpectedDiscomfort_{Sars} \\ & - P_{A1}(Dose) \times DW_M \\ & - P_{A3+}(Dose) \times DW_S \\ & - 0.655 \times P_{S'}(Dose) \times ExpectedDiscomfort_{Sars} \end{aligned}$$

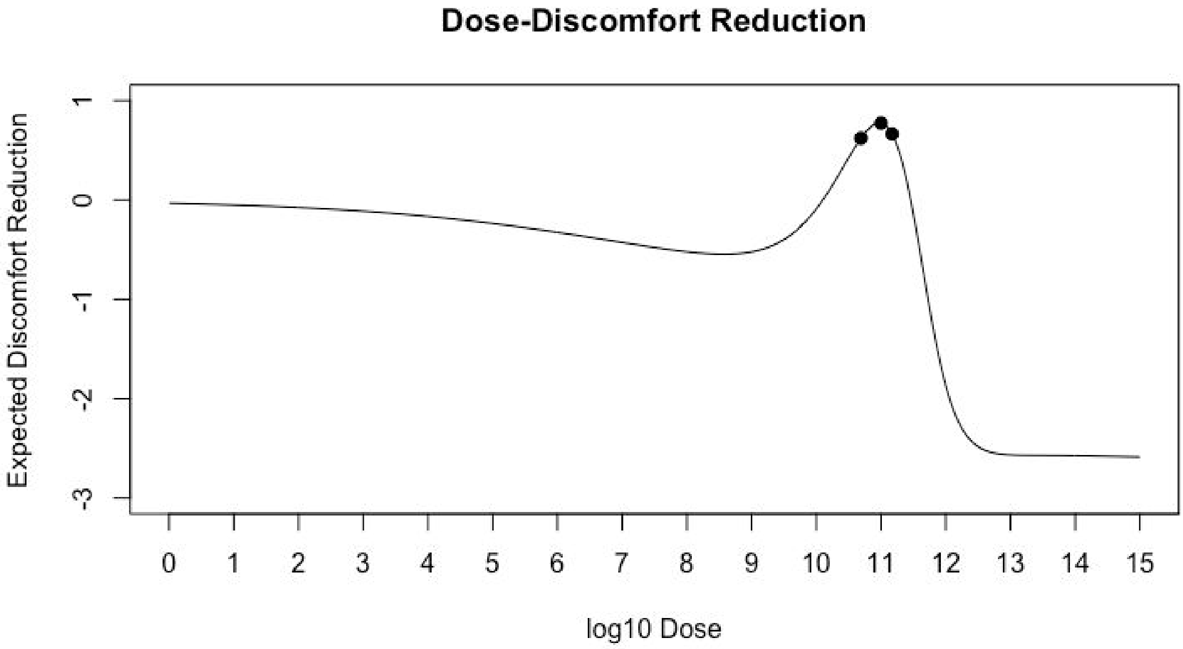

Figure S15. Baseline subtracted Dose-Utility for the expected discomfort utility function. Black dots represent the empirically tested doses.

Further, we can consider only the doses where the discomfort reduction is predicted to be greater than 0. Hence, by dividing by the 'dose-cost' model found in the main body of this work we can also estimate the expected reduction in discomfort per GBP spent on the vaccine at each dosing level.

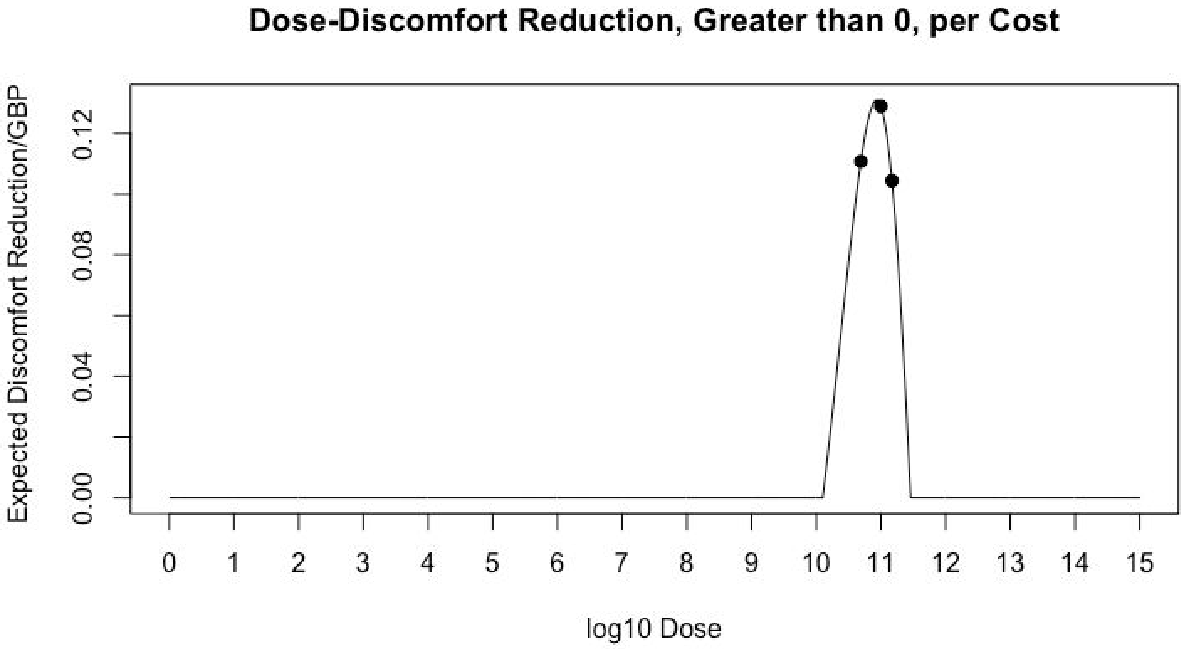

Figure S16. Baseline subtracted Dose-Utility for the expected discomfort utility function, divided by cost and censored if discomfort reduction is less than 0. Black dots represent the empirically tested doses.
